# Supplementary material for: Phage therapy for recurrent urinary tract infections: A qualitative study using the theoretical framework of acceptability
Source: PLoS One. 2026 May 19;21(5):e0349568. doi: 10.1371/journal.pone.0349568 (PMC13186363; doi:10.1371/journal.pone.0349568)
Supplement: S1 Fig — (PDF) [file pone.0349568.s001.pdf]

Group	Overall Theme	Questions	Prompts	
**Individuals with recurrent UTIs**	Existing knowledge of PT as a treatment.	**What information do you know about PT?**	Other than today, what have you heard about relating to PT? In what context?	Describe initial reactions to the use of PT
	Personal perceptions, feelings and attitudes to being prescribed with PT as a treatment for UTIs.	**What would influence your personal decision to be treated with PT?**	What would be a barrier to you using this yourself?	Would the way the treatment is offered make a difference (e.g., route of administration?)
	Barriers and enablers to individuals being treated with PT.	**Describe barriers to PT that might exist in the population?**	How has the pandemic made a difference to how people feel?	How does this type of approach to treatment sound like any other medical way to target bacteria / germs etc.<br>To what extent do you feel the approach sounds modern and forward thinking?
	Challenging myths.	**Describe a situation where you might be prepared to use PT yourself (or a time when you would refuse it as an approach)**	What would help you feel comfortable about being prescribed PT?	What situations are there where you wouldn't want to use it? (e.g., certain times of life; different ages)

Group	Overall Theme	Questions	Prompts	
**Doctors**	Existing knowledge of PT as a treatment.	**What information do you know about PT?**	Other than today, what have you heard about relating to PT? In what context?	How has the way the information was given to you, affected your opinions on using PT?
	Attitudes to treated with PT for UTIs.	**What do you think would be patients' views on using PT as a treatment?**	What might be the barriers and enablers for patients in using PT?	Would the way the treatment is offered make a difference (e.g., route of administration?)<br>How has the pandemic made a difference to how you might prescribe PT?
	Barriers and enablers to prescribing PT.	**What do you think would be the doctors' views on using PT as a treatment for their patients?**	Attitudes if you were to prescribe to someone in your own family?	How would you feel about being treated yourself with PT?
	Challenging myths.	**Who should take responsibility?**	Who should prescribe PT? e.g., specialist physician or GP?	How would GPs feel about prescribing PT?

Group	Overall Theme	Questions	Prompts	
**Other Clinicians / Healthcare professionals**	Existing knowledge of PT as a treatment for UTIs	**What information do you know about PT?**	Other than today, what have you heard about relating to PT? In what context?	How has the way the information was given to you, affected your opinions on using PT?
	Attitudes to treated with PT for UTIs.<br><br>Barriers and enablers that might exist in patients.	**What do you think would be patients' views on using PT as a treatment?**	What might be the barriers and enablers for patients in using PT?<br><br>How would you feel about being treated yourself with PT?	How has the pandemic made a difference to how you might feel about patients being prescribed PT?<br><br>Based on your understanding of PT from the information provided, what may prevent you from taking PT as a treatment option?
	Challenging myths.	**Describe a situation where you might be prepared to use PT yourself (or a time when you would refuse it as an approach)**	What would help you feel comfortable about being prescribed PT?	Any situations where you wouldn't want to use it? (e.g., certain times of life; different ages)
