# Supplementary material for: Phage therapy for recurrent urinary tract infections: A qualitative study using the theoretical framework of acceptability
Source: PLoS One. 2026 May 19;21(5):e0349568. doi: 10.1371/journal.pone.0349568 (PMC13186363; doi:10.1371/journal.pone.0349568)
Supplement: S2 Fig — Table 2: Number of participants per stakeholder group, country of origin and gender. (DOCX) [file pone.0349568.s002.docx]

### S2 Fig

Table 1 – Date and times of focus groups, stakeholder group, number of participants and length where recorded.

| Group Date | Participants | Numbers | Length (minutes) |
| --- | --- | --- | --- |
| 17 June 2024 at 1.00 - 2.30 pm | Nurse & AHP | 10 |  |
| 18 June 2024 at 2.00 - 3.30 pm | Lived Experience | 9 | 72 |
| 19 June 2024 at 3.00 - 4.30 pm | Nurse & AHP | 8 |  |
| 20 June 2024 at 1.00 -2.30 pm | Lived Experience | 9 | 66 |
| 25 June 2024 at 5.30 -7.00 pm | DR | 9 |  |
| 28 June 2024 at 10.30 am -12.00 pm | DR | 9 |  |

Focus Group Details

Table 2 – Number of participants per stakeholder group, country of origin and gender

| **Group** | **Number** | **Country** |  | **Gender** |  |
| --- | --- | --- | --- | --- | --- |
| Healthcare Professionals  Nurses-14  Pharmacist -3  Not stated - 1 | 18 | UK (Not Stated country) | 10 | Male | 8 |
|  |  | Scotland | 3 |  |  |
|  |  | England | 5 | Female | 10 |
|  |  | Wales | 0 |  |  |
| Drs | 18 | UK (Not Stated country) | 10 | Male | 16 |
|  |  | Scotland | 1 |  |  |
|  |  | England | 5 | Female | 2 |
|  |  | Wales | 2 |  |  |
| Lived Experience | 19 | UK (Not Stated country) | 12 | Male | 6 |
|  |  | Scotland |  |  |  |
|  |  | England | 6 | Female | 13 |
|  |  | Wales |  |  |  |

Characteristics of Participants
