# Supplementary material for: Phage therapy for recurrent urinary tract infections: A qualitative study using the theoretical framework of acceptability
Source: PLoS One. 2026 May 19;21(5):e0349568. doi: 10.1371/journal.pone.0349568 (PMC13186363; doi:10.1371/journal.pone.0349568)
Supplement: Attachment — Submitted filename: Response to Reviewers.docx [file pone.0349568.s004.docx]

Journal Requirements:

When submitting your revision, we need you to address these additional requirements.

1.Please ensure that your manuscript meets PLOS ONE's style requirements, including those for file naming. The PLOS ONE style templates can be found at

<https://journals.plos.org/plosone/s/file?id=wjVg/PLOSOne_formatting_sample_main_body.pdf> and

The header orders have been upscaled, the spacing increased to double line spacing, references have been updated to match preferred format.

[https://journals.plos.org/plosone/s/file?id=ba62/PLOSOne_formatting_sample_title_authors_affiliations.pdf](https://eur03.safelinks.protection.outlook.com/?url=https%3A%2F%2Ftrack.editorialmanager.com%2FCL0%2Fhttps%3A%252F%252Fjournals.plos.org%252Fplosone%252Fs%252Ffile%253Fid%3Dba62%252FPLOSOne_formatting_sample_title_authors_affiliations.pdf%2F1%2F010f019bd586126f-72d98323-19b3-411c-988b-ad29ea8f8cab-000000%2Fj9UgsnJsm1v6bGKHmrL2rEycO7wAfFzUeLw6c71Q_UE%3D246&data=05%7C02%7Cdw343%40leicester.ac.uk%7C6e52b26deeaa40417e8508de573abdbe%7Caebecd6a31d44b0195ce8274afe853d9%7C0%7C1%7C639044106978319416%7CUnknown%7CTWFpbGZsb3d8eyJFbXB0eU1hcGkiOnRydWUsIlYiOiIwLjAuMDAwMCIsIlAiOiJXaW4zMiIsIkFOIjoiTWFpbCIsIldUIjoyfQ%3D%3D%7C0%7C%7C%7C&sdata=6VB%2BJEhAjqpG%2Fe51F%2FmFbpBaid8Widt4WBRmJTU7r%2Bg%3D&reserved=0)

The title page has been extensively reformatted

2. Please note that funding information should not appear in any section or other areas of your manuscript. We will only publish funding information present in the Funding Statement section of the online submission form. Please remove any funding-related text from the manuscript.

All funding related text has been removed.

3. Please expand the acronym “NIHR” (as indicated in your financial disclosure) so that it states the name of your funders in full.

This information should be included in your cover letter; we will change the online submission form on your behalf.

Thank you, the funding statement is now removed from the manuscript and the funding information section is in full. I will ensure the funding is included within the new cover letter.

4. In the online submission form you indicate that your data is not available for proprietary reasons and have provided a contact point for accessing this data. Please note that your current contact point is a co-author on this manuscript. According to our Data Policy, the contact point must not be an author on the manuscript and must be an institutional contact, ideally not an individual. Please revise your data statement to a non-author institutional point of contact, such as a data access or ethics committee, and send this to us via return email. Please also include contact information for the third party organization, and please include the full citation of where the data can be found.

Many thanks, the data availability statement has been updated.

5. Your ethics statement should only appear in the Methods section of your manuscript. If your ethics statement is written in any section besides the Methods, please move it to the Methods section and delete it from any other section. Please ensure that your ethics statement is included in your manuscript, as the ethics statement entered into the online submission form will not be published alongside your manuscript.

The reformatting of the headers now ensures the ethics statement is included within the methods section.

6. Please remove all personal information/transcripts within your manuscript, ensure that the data shared are in accordance with participant consent, and re-upload a fully anonymized data set.

Additional guidance on preparing raw data for publication can be found in our Data Policy ([https://journals.plos.org/plosone/s/data-availability#loc-human-research-participant-data-and-other-sensitive-data](https://eur03.safelinks.protection.outlook.com/?url=https%3A%2F%2Ftrack.editorialmanager.com%2FCL0%2Fhttps%3A%252F%252Fjournals.plos.org%252Fplosone%252Fs%252Fdata-availability%2523loc-human-research-participant-data-and-other-sensitive-data%2F1%2F010f019bd586126f-72d98323-19b3-411c-988b-ad29ea8f8cab-000000%2FDQLEO5ypdUnenyCRo9Xag_7-YdTqAPQqSR0scyglqI8%3D246&data=05%7C02%7Cdw343%40leicester.ac.uk%7C6e52b26deeaa40417e8508de573abdbe%7Caebecd6a31d44b0195ce8274afe853d9%7C0%7C1%7C639044106978334671%7CUnknown%7CTWFpbGZsb3d8eyJFbXB0eU1hcGkiOnRydWUsIlYiOiIwLjAuMDAwMCIsIlAiOiJXaW4zMiIsIkFOIjoiTWFpbCIsIldUIjoyfQ%3D%3D%7C0%7C%7C%7C&sdata=dY%2BYoRx94kT7dBW%2FfC4Qapco728DDiKpBpZ59hmS1fo%3D&reserved=0)) and in the following article: [http://www.bmj.com/content/340/bmj.c181.long](https://eur03.safelinks.protection.outlook.com/?url=http%3A%2F%2Ftrack.editorialmanager.com%2FCL0%2Fhttp%3A%252F%252Fwww.bmj.com%252Fcontent%252F340%252Fbmj.c181.long%2F1%2F010f019bd586126f-72d98323-19b3-411c-988b-ad29ea8f8cab-000000%2FlXt6bHdg9PG2QmlcaIpS6lsY_SRYlwEYWC-g4_StWMc%3D246&data=05%7C02%7Cdw343%40leicester.ac.uk%7C6e52b26deeaa40417e8508de573abdbe%7Caebecd6a31d44b0195ce8274afe853d9%7C0%7C1%7C639044106978350252%7CUnknown%7CTWFpbGZsb3d8eyJFbXB0eU1hcGkiOnRydWUsIlYiOiIwLjAuMDAwMCIsIlAiOiJXaW4zMiIsIkFOIjoiTWFpbCIsIldUIjoyfQ%3D%3D%7C0%7C%7C%7C&sdata=kWpEMElACUOVi4Bi0Pa6%2BGL01GRJ8BNgE5Qrh3YPIuQ%3D&reserved=0).

Thank you for the guidance and we appreciate the links provided. We would like to clarify our understanding and ensure full compliance with PLOS ONE’s data policy.

The manuscript contains only selected illustrative quotations, which have been anonymised using non-identifying participant labels (e.g. stakeholder group and coded participant identifier) and do not include names, locations, institutions, or other potentially identifying information. No full transcripts or raw qualitative datasets are included within the manuscript or its appendices. Please can you clarify if there are areas of potential concern around identification of participants?

7. If the reviewer comments include a recommendation to cite specific previously published works, please review and evaluate these publications to determine whether they are relevant and should be cited. There is no requirement to cite these works unless the editor has indicated otherwise. 

Many thanks.

[Note: HTML markup is below. Please do not edit.]

Reviewers' comments:

Reviewer's Responses to Questions

**Comments to the Author**

1. Is the manuscript technically sound, and do the data support the conclusions?

The manuscript must describe a technically sound piece of scientific research with data that supports the conclusions. Experiments must have been conducted rigorously, with appropriate controls, replication, and sample sizes. The conclusions must be drawn appropriately based on the data presented.

Reviewer #1: Yes

Reviewer #2: Partly

2. Has the statistical analysis been performed appropriately and rigorously?

Reviewer #1: N/A

Reviewer #2: N/A

3. Have the authors made all data underlying the findings in their manuscript fully available?

The [PLOS Data policy](https://eur03.safelinks.protection.outlook.com/?url=http%3A%2F%2Ftrack.editorialmanager.com%2FCL0%2Fhttp%3A%252F%252Fwww.plosone.org%252Fstatic%252Fpolicies.action%2523sharing%2F1%2F010f019bd586126f-72d98323-19b3-411c-988b-ad29ea8f8cab-000000%2Fyn20CCHYUeyd_m8-AZQgUY-RS6QqgZ42LaHNmWh6OMM%3D246&data=05%7C02%7Cdw343%40leicester.ac.uk%7C6e52b26deeaa40417e8508de573abdbe%7Caebecd6a31d44b0195ce8274afe853d9%7C0%7C1%7C639044106978365613%7CUnknown%7CTWFpbGZsb3d8eyJFbXB0eU1hcGkiOnRydWUsIlYiOiIwLjAuMDAwMCIsIlAiOiJXaW4zMiIsIkFOIjoiTWFpbCIsIldUIjoyfQ%3D%3D%7C0%7C%7C%7C&sdata=%2F3J6lPNrM7SexNK5iHWXujlZuxjQ6WdqorJKQ7qhMk4%3D&reserved=0) requires authors to make all data underlying the findings described in their manuscript fully available without restriction, with rare exception (please refer to the Data Availability Statement in the manuscript PDF file). The data should be provided as part of the manuscript or its supporting information, or deposited to a public repository. For example, in addition to summary statistics, the data points behind means, medians and variance measures should be available. If there are restrictions on publicly sharing data—e.g. participant privacy or use of data from a third party—those must be specified.

Reviewer #1: Yes

Reviewer #2: Yes

4. Is the manuscript presented in an intelligible fashion and written in standard English?

PLOS ONE does not copyedit accepted manuscripts, so the language in submitted articles must be clear, correct, and unambiguous. Any typographical or grammatical errors should be corrected at revision, so please note any specific errors here.

Reviewer #1: Yes

Reviewer #2: Yes

5. Review Comments to the Author

Please use the space provided to explain your answers to the questions above. You may also include additional comments for the author, including concerns about dual publication, research ethics, or publication ethics. (Please upload your review as an attachment if it exceeds 20,000 characters)

Reviewer #1: This manuscript presents a rigorous, well-executed qualitative study exploring the anticipated acceptability of bacteriophage therapy for recurrent urinary tract infections using the Theoretical Framework of Acceptability. The topic is timely, ethically important, and well suited to PLOS ONE’s scope. The manuscript is clearly written, methodologically sound, and demonstrates strong engagement with both theory and empirical data.

Minor points for improvement

1. Reflexivity
While reflexive thematic analysis is cited, a brief reflexivity statement (e.g., researchers’ disciplinary backgrounds or relationship to phage research) would further strengthen methodological transparency.

Many thanks for this helpful comment. A reflexive thematic analysis approach was adopted, and we agree that a brief reflexivity statement strengthens methodological transparency. We had previously kept this section minimal to preserve manuscript flow, but have now reintroduced a concise reflexivity statement that remains at a team level rather than providing a detailed positionality account for individual authors. We believe this strikes an appropriate balance between clarity, reflexivity, and readability (added to the end of methods).

2. Online community findings
The discussion of online peer communities is insightful. The authors may wish to clarify more explicitly that this theme emerged inductively and sits partially outside the TFA, as they note, to avoid any perception of conceptual overextension.

We have clarified that the theme relating to online peer communities emerged inductively during analysis and is presented as an interpretive finding that sits partially outside the TFA, to avoid any implication of conceptual overextension (line added in discussion).

3. Terminology consistency
Minor improvements could be made by ensuring consistent terminology when referring to stakeholder groups (e.g., “doctors” vs. “clinicians”) throughout the manuscript.

Many thanks for this helpful suggestion. The term “clinicians” was originally used to collectively refer to doctors and other healthcare professionals; however, on review we agreed that this occasionally obscured attribution to specific stakeholder groups. We have therefore removed use of the collective term and clarified throughout whether findings relate to doctors, healthcare professionals, or both.

Reviewer #2: Good, overall. This manuscript does a nice job of introducing the TFA model and how it relates to phage therapy here. The discussion section aptly summarizes the anecdotal statements made by the 6 online focus groups. It is clear that desperate rUTI patients are most likely to view phage therapy as an appropriate treatment; it is also evident that financial cost and the lack of knowledge surrounding phage therapy are clear deterrents. I also appreciate that the study's limitations were elaborated upon. Furthermore, the entire manuscript is well-written.

However, I am a bit concerned about the lack of quantitative analyses done here, with respect to affective attitude, perceived effectiveness, ethicality, burden, opportunity costs, etc. Is there a way to quantify the responses of the 3 stakeholder groups and how they differ across the 7 constructs of the TFA? That way, the reader would be more readily able to determine how, for instance, doctors and healthcare professionals (i.e.-2 of the 3 stakeholder groups detailed here) differ in their viewpoints of phage therapy's burden, opportunity costs, ethics, etc. Although anecdotal statements from the focus groups, as done here, are somewhat insightful, quantitative analyses would prove to be even more revealing. For example, the authors could parse through the focus group discussions and quantify the frequency of statements associated with "positive affection" or "negative affection" for the "Affective Attitude" arm of the TFA. Alternatively, if the authors asked the respondents to rank perceived effectiveness on a quantitative scale, this would allow us to understand how the 3 different stakeholder groups differ on the basis of concrete numerical mean differences (e.g.-a "perceived effectiveness" mean score for each group). All in all, there are many ways that the authors could add a quantitative dimension to this study, addressing the 7 constructs of the TFA across the 3 stakeholder groups. This would be greatly appreciated.

We thank the reviewer for this thoughtful comment and for highlighting the potential value of quantitative approaches to examining acceptability across TFA constructs.

The aim of this study was to explore anticipated acceptability qualitatively using focus groups and reflexive thematic analysis (RTA), rather than to quantify differences between stakeholder groups. Consistent with the philosophical and epistemological underpinnings of RTA, frequency counts or numerical comparisons were not planned, as such approaches do not align with the interpretive focus of this analytic method and may misrepresent the salience or meaning of themes within interactive group discussions.

To make this methodological position clearer, we have now explicitly stated in the Methods section that no quantitative analysis was planned or undertaken, in line with Braun and Clarke’s guidance on reflexive thematic analysis. We have also clarified in the Limitations section that while the findings may inform future quantitative or mixed-methods research examining acceptability across TFA constructs, such analyses were beyond the scope of this paper.

6. PLOS authors have the option to publish the peer review history of their article ([what does this mean?](https://eur03.safelinks.protection.outlook.com/?url=https%3A%2F%2Ftrack.editorialmanager.com%2FCL0%2Fhttps%3A%252F%252Fjournals.plos.org%252Fplosone%252Fs%252Feditorial-and-peer-review-process%2523loc-peer-review-history%2F1%2F010f019bd586126f-72d98323-19b3-411c-988b-ad29ea8f8cab-000000%2FN9RevvrvYOC3XnePxtdfadpzfFuNx6Zor-7BhKb6OnM%3D246&data=05%7C02%7Cdw343%40leicester.ac.uk%7C6e52b26deeaa40417e8508de573abdbe%7Caebecd6a31d44b0195ce8274afe853d9%7C0%7C1%7C639044106978380628%7CUnknown%7CTWFpbGZsb3d8eyJFbXB0eU1hcGkiOnRydWUsIlYiOiIwLjAuMDAwMCIsIlAiOiJXaW4zMiIsIkFOIjoiTWFpbCIsIldUIjoyfQ%3D%3D%7C0%7C%7C%7C&sdata=C6esLm2LPYUG109jys84Cph31VY%2FhoCW%2FWThHO2iF1g%3D&reserved=0)). If published, this will include your full peer review and any attached files.


If you choose “no”, your identity will remain anonymous but your review may still be made public.


**Do you want your identity to be public for this peer review?** For information about this choice, including consent withdrawal, please see our [Privacy Policy](https://eur03.safelinks.protection.outlook.com/?url=https%3A%2F%2Ftrack.editorialmanager.com%2FCL0%2Fhttps%3A%252F%252Fwww.plos.org%252Fprivacy-policy%2F1%2F010f019bd586126f-72d98323-19b3-411c-988b-ad29ea8f8cab-000000%2FuHEyC5rTndnZ9q1RFaOMFms7NM6hyoK9PEdgQnKymkk%3D246&data=05%7C02%7Cdw343%40leicester.ac.uk%7C6e52b26deeaa40417e8508de573abdbe%7Caebecd6a31d44b0195ce8274afe853d9%7C0%7C1%7C639044106978393790%7CUnknown%7CTWFpbGZsb3d8eyJFbXB0eU1hcGkiOnRydWUsIlYiOiIwLjAuMDAwMCIsIlAiOiJXaW4zMiIsIkFOIjoiTWFpbCIsIldUIjoyfQ%3D%3D%7C0%7C%7C%7C&sdata=m5yKbV25hKJCCwQWgFY%2BNRNI0sBXQomuabmhvFq5ugc%3D&reserved=0).

Reviewer #1: **Yes:** Ali Bin Thani

Reviewer #2: No
