# Supplementary material for: Phage therapy for recurrent urinary tract infections: A qualitative study using the theoretical framework of acceptability
Source: PLoS One. 2026 May 19;21(5):e0349568. doi: 10.1371/journal.pone.0349568 (PMC13186363; doi:10.1371/journal.pone.0349568)
Supplement: Attachment — Submitted filename: Response_to_Reviewers_auresp_2.docx [file pone.0349568.s005.docx]

Dear Dr. Wenzel,

Thank you for submitting your manuscript to PLOS ONE. After careful consideration, we feel that it has merit but does not fully meet PLOS ONE’s publication criteria as it currently stands. Therefore, we invite you to submit a revised version of the manuscript that addresses the points raised during the review process.

Please submit your revised manuscript by Apr 17 2026 11:59PM. If you will need more time than this to complete your revisions, please reply to this message or contact the journal office at [plosone@plos.org](mailto:plosone@plos.org). When you're ready to submit your revision, log on to https://www.editorialmanager.com/pone/ and select the 'Submissions Needing Revision' folder to locate your manuscript file.

Please include the following items when submitting your revised manuscript:

- A letter that responds to each point raised by the academic editor and reviewer(s). You should upload this letter as a separate file labeled 'Response to Reviewers'.
- A marked-up copy of your manuscript that highlights changes made to the original version. You should upload this as a separate file labeled 'Revised Manuscript with Track Changes'.
- An unmarked version of your revised paper without tracked changes. You should upload this as a separate file labeled 'Manuscript'.

If you would like to make changes to your financial disclosure, please include your updated statement in your cover letter. Guidelines for resubmitting your figure files are available below the reviewer comments at the end of this letter.

If applicable, we recommend that you deposit your laboratory protocols in protocols.io to enhance the reproducibility of your results. Protocols.io assigns your protocol its own identifier (DOI) so that it can be cited independently in the future. For instructions see: [https://journals.plos.org/plosone/s/submission-guidelines#loc-laboratory-protocols](https://eur03.safelinks.protection.outlook.com/?url=https%3A%2F%2Ftrack.editorialmanager.com%2FCL0%2Fhttps%3A%252F%252Fjournals.plos.org%252Fplosone%252Fs%252Fsubmission-guidelines%2523loc-laboratory-protocols%2F1%2F010f019cb22ede43-3c85384f-0716-4ec2-909a-60fff15c30f5-000000%2FIopfI9xtAhlahUG9S0YDPrGnNpknhj5hljHVbtCQ-Vw%3D252&data=05%7C02%7Cdw343%40leicester.ac.uk%7C486d8fa1b49848f67c5908de78e63f4c%7Caebecd6a31d44b0195ce8274afe853d9%7C0%7C0%7C639081127473534564%7CUnknown%7CTWFpbGZsb3d8eyJFbXB0eU1hcGkiOnRydWUsIlYiOiIwLjAuMDAwMCIsIlAiOiJXaW4zMiIsIkFOIjoiTWFpbCIsIldUIjoyfQ%3D%3D%7C0%7C%7C%7C&sdata=oy6vV%2FBamw1CbvEvp67c8669d0n2Gxs5hcrYbqTrxSc%3D&reserved=0). Additionally, PLOS ONE offers an option for publishing peer-reviewed Lab Protocol articles, which describe protocols hosted on protocols.io. Read more information on sharing protocols at [https://plos.org/protocols?utm_medium=editorial-email&utm_source=authorletters&utm_campaign=protocols](https://eur03.safelinks.protection.outlook.com/?url=https%3A%2F%2Ftrack.editorialmanager.com%2FCL0%2Fhttps%3A%252F%252Fplos.org%252Fprotocols%253Futm_medium%3Deditorial-email%2526utm_source%3Dauthorletters%2526utm_campaign%3Dprotocols%2F1%2F010f019cb22ede43-3c85384f-0716-4ec2-909a-60fff15c30f5-000000%2F8xNqVwv-icOp_UKrJkd9oaUMOv_m1KVMR9dOa0DsEzI%3D252&data=05%7C02%7Cdw343%40leicester.ac.uk%7C486d8fa1b49848f67c5908de78e63f4c%7Caebecd6a31d44b0195ce8274afe853d9%7C0%7C0%7C639081127473548364%7CUnknown%7CTWFpbGZsb3d8eyJFbXB0eU1hcGkiOnRydWUsIlYiOiIwLjAuMDAwMCIsIlAiOiJXaW4zMiIsIkFOIjoiTWFpbCIsIldUIjoyfQ%3D%3D%7C0%7C%7C%7C&sdata=AEIbPucol2fnvuzQRjAAoqMRgoSgfymMPfhL%2BbuGrVw%3D&reserved=0).

We look forward to receiving your revised manuscript.

Kind regards,

Awatif Abid Al-Judaibi, PhD
Academic Editor
PLOS One

Journal Requirements:

1. If the reviewer comments include a recommendation to cite specific previously published works, please review and evaluate these publications to determine whether they are relevant and should be cited. There is no requirement to cite these works unless the editor has indicated otherwise. 

2. Please review your reference list to ensure that it is complete and correct. If you have cited papers that have been retracted, please include the rationale for doing so in the manuscript text, or remove these references and replace them with relevant current references. Any changes to the reference list should be mentioned in the rebuttal letter that accompanies your revised manuscript. If you need to cite a retracted article, indicate the article’s retracted status in the References list and also include a citation and full reference for the retraction notice.

[Note: HTML markup is below. Please do not edit.]

Reviewers' comments:

Reviewer's Responses to Questions

**Comments to the Author**

1. If the authors have adequately addressed your comments raised in a previous round of review and you feel that this manuscript is now acceptable for publication, you may indicate that here to bypass the “Comments to the Author” section, enter your conflict of interest statement in the “Confidential to Editor” section, and submit your "Accept" recommendation.

Reviewer #2: All comments have been addressed

Reviewer #3: All comments have been addressed

Reviewer #4: (No Response)

2. Is the manuscript technically sound, and do the data support the conclusions?

The manuscript must describe a technically sound piece of scientific research with data that supports the conclusions. Experiments must have been conducted rigorously, with appropriate controls, replication, and sample sizes. The conclusions must be drawn appropriately based on the data presented.

Reviewer #2: (No Response)

Reviewer #3: Yes

Reviewer #4: Partly

3. Has the statistical analysis been performed appropriately and rigorously?

Reviewer #2: (No Response)

Reviewer #3: Yes

Reviewer #4: Yes

4. Have the authors made all data underlying the findings in their manuscript fully available?

The [PLOS Data policy](https://eur03.safelinks.protection.outlook.com/?url=http%3A%2F%2Ftrack.editorialmanager.com%2FCL0%2Fhttp%3A%252F%252Fwww.plosone.org%252Fstatic%252Fpolicies.action%2523sharing%2F1%2F010f019cb22ede43-3c85384f-0716-4ec2-909a-60fff15c30f5-000000%2FuOXIh3tSw6uCgOUJ2Os5OyRmX9BBXkztAjhsWsmrca4%3D252&data=05%7C02%7Cdw343%40leicester.ac.uk%7C486d8fa1b49848f67c5908de78e63f4c%7Caebecd6a31d44b0195ce8274afe853d9%7C0%7C0%7C639081127473556799%7CUnknown%7CTWFpbGZsb3d8eyJFbXB0eU1hcGkiOnRydWUsIlYiOiIwLjAuMDAwMCIsIlAiOiJXaW4zMiIsIkFOIjoiTWFpbCIsIldUIjoyfQ%3D%3D%7C0%7C%7C%7C&sdata=8qMiMsqS4zMIXItkPynMvVSEVYGNGUlA1iM87QnJjtA%3D&reserved=0) requires authors to make all data underlying the findings described in their manuscript fully available without restriction, with rare exception (please refer to the Data Availability Statement in the manuscript PDF file). The data should be provided as part of the manuscript or its supporting information, or deposited to a public repository. For example, in addition to summary statistics, the data points behind means, medians and variance measures should be available. If there are restrictions on publicly sharing data—e.g. participant privacy or use of data from a third party—those must be specified.

Reviewer #2: (No Response)

Reviewer #3: Yes

Reviewer #4: Yes

5. Is the manuscript presented in an intelligible fashion and written in standard English?

PLOS ONE does not copyedit accepted manuscripts, so the language in submitted articles must be clear, correct, and unambiguous. Any typographical or grammatical errors should be corrected at revision, so please note any specific errors here.

Reviewer #2: (No Response)

Reviewer #3: Yes

Reviewer #4: Yes

6. Review Comments to the Author

Please use the space provided to explain your answers to the questions above. You may also include additional comments for the author, including concerns about dual publication, research ethics, or publication ethics. (Please upload your review as an attachment if it exceeds 20,000 characters)

Reviewer #2: (No Response)

Reviewer #3: (No Response)

Reviewer #4: This manuscript presents a qualitative study exploring stakeholder perceptions regarding the anticipated acceptability of bacteriophage therapy for recurrent urinary tract infections using the Theoretical Framework of Acceptability. The qualitative design and use of focus groups are appropriate for the stated aims, and the conclusions are generally supported by the data presented. The manuscript is clearly structured overall; however, several issues should be addressed to improve methodological transparency and reporting quality.

First, recruitment through social media and networks connected to phage research may have introduced selection bias toward participants already interested in novel therapies. The implications of this for transferability of findings should be more explicitly discussed.

We thank the reviewer for this comment. The Limitations section already acknowledged the implications of recruitment through phage-related networks for transferability. We have now revised this paragraph to make this discussion more explicit and to provide additional analytic depth.

In addition, further clarification is needed regarding how “information power” was determined…

We thank the reviewer for this helpful comment. We agree that greater methodological transparency was warranted. Unfortunately, some of this more granular detail was removed for length, but we are glad to now be able to include it.
 We have revised the Methods section to provide a clearer account of how information power was assessed and how the analytic process proceeded. Specifically, we now clarify that information power was reviewed iteratively during data collection, guided by Malterud et al.’s paper. We had two study committee meetings regarding information power, after the first 4 focus groups and after all 6 had been completed. In these meetings we assessed the components of information power that were not known a priori including specificity, dialogue and alignment with the theoretical framework.

… and how inductive coding was subsequently mapped onto TFA constructs, as this process is currently insufficiently described.

We have additionally addressed the transparency of our coding process. The Data Analysis section has been re-written in full to create a more coherent, easy to follow process. Rather than addressing analysis first and information power second the two constructs are addressed alongside one another to plot better against the data analysis meetings that were held. We believe this makes the process easier to follow whilst increasing transparency.

Second, the Data Availability Statement indicates that data are available only upon reasonable request. This does not appear fully aligned with PLOS ONE data availability requirements unless justified ethical restrictions apply. The authors should clarify whether anonymized qualitative data can be deposited in a repository or provide a clearer explanation of access procedures and restrictions.

We thank the reviewer for this important point.

The qualitative dataset contains detailed personal and professional accounts relating to clinical practice and lived health experiences. Although transcripts were anonymised, the depth and contextual specificity of the discussions mean that full public deposition would carry a risk of deductive disclosure. Ethical approval for the study was granted on the basis that data would be stored securely and accessed under controlled conditions rather than deposited in an open repository.

We have discussed the Data Availability Statement with the journal office during the previous revision round, and the current approach was confirmed to be consistent with PLOS ONE policy for qualitative research involving sensitive data. We have now revised the wording to provide a clearer explanation of the access procedures and restrictions, including institutional contact details and governance requirements for researchers seeking access to additional anonymised material.

We hope this clarification addresses the reviewer’s concern.

Several presentation issues also require attention. Some references cited in the Introduction (e.g., early epidemiological statements) appear to rely on secondary sources rather than original studies and should be revised where appropriate. Referencing style is inconsistent throughout the manuscript and should be standardized according to journal guidelines. Tables currently lack clear descriptive titles and should be revised so they can be interpreted independently of the main text. Finally, formatting artifacts and editing marks visible in the manuscript should be removed prior to publication.

We thank the reviewer for these helpful observations.

We have revised the Introduction to ensure that early epidemiological statements are supported by appropriate primary sources, including replacing the citation of Germanos et al. with Schaeffer et al. where this more directly reflects the original evidence.

The reference list has been carefully reviewed and standardised in accordance with PLOS ONE guidance, using Vancouver style consistently throughout. Formatting inconsistencies introduced during manuscript preparation have now been corrected to ensure uniform presentation.

Table titles have been revised to provide clearer, more descriptive headings so that each table can be interpreted independently of the main text.

The manuscript has also been reviewed in full to remove formatting artefacts and ensure that tracked changes are fully resolved prior to submission.

7. PLOS authors have the option to publish the peer review history of their article ([what does this mean?](https://eur03.safelinks.protection.outlook.com/?url=https%3A%2F%2Ftrack.editorialmanager.com%2FCL0%2Fhttps%3A%252F%252Fjournals.plos.org%252Fplosone%252Fs%252Feditorial-and-peer-review-process%2523loc-peer-review-history%2F1%2F010f019cb22ede43-3c85384f-0716-4ec2-909a-60fff15c30f5-000000%2FoGzlfTpBq_QVUwcvw_mIK82W0n7CrxUqyyfx1r0LHhw%3D252&data=05%7C02%7Cdw343%40leicester.ac.uk%7C486d8fa1b49848f67c5908de78e63f4c%7Caebecd6a31d44b0195ce8274afe853d9%7C0%7C0%7C639081127473564501%7CUnknown%7CTWFpbGZsb3d8eyJFbXB0eU1hcGkiOnRydWUsIlYiOiIwLjAuMDAwMCIsIlAiOiJXaW4zMiIsIkFOIjoiTWFpbCIsIldUIjoyfQ%3D%3D%7C0%7C%7C%7C&sdata=cjG1KxVvtXyoVosIACnH2HyamEEn4xKTwUHUdIzcsDo%3D&reserved=0)). If published, this will include your full peer review and any attached files.


If you choose “no”, your identity will remain anonymous but your review may still be made public.


**Do you want your identity to be public for this peer review?** For information about this choice, including consent withdrawal, please see our [Privacy Policy](https://eur03.safelinks.protection.outlook.com/?url=https%3A%2F%2Ftrack.editorialmanager.com%2FCL0%2Fhttps%3A%252F%252Fwww.plos.org%252Fprivacy-policy%2F1%2F010f019cb22ede43-3c85384f-0716-4ec2-909a-60fff15c30f5-000000%2FFP2mL-J5vuCJq8gw7rxYhFHdOwG_xCH6jHH0e7VwlTk%3D252&data=05%7C02%7Cdw343%40leicester.ac.uk%7C486d8fa1b49848f67c5908de78e63f4c%7Caebecd6a31d44b0195ce8274afe853d9%7C0%7C0%7C639081127473572151%7CUnknown%7CTWFpbGZsb3d8eyJFbXB0eU1hcGkiOnRydWUsIlYiOiIwLjAuMDAwMCIsIlAiOiJXaW4zMiIsIkFOIjoiTWFpbCIsIldUIjoyfQ%3D%3D%7C0%7C%7C%7C&sdata=hjQj7oozpHFv0t8EgGqZVn5jFTetipL1qEL1Kz%2BqsqM%3D&reserved=0).

Reviewer #2: No

Reviewer #3: No

Reviewer #4: **Yes:** Zaid Iyad Alkhatib
